# Pilots, Astronauts, and the Aerospace Microbiota: A Narrative Review of Occupational Impact

**DOI:** 10.7759/cureus.72268

**Published:** 2024-10-24

**Authors:** Piercarlo Minoretti, Jacopo M Fontana, Yusuf Yilmaz

**Affiliations:** 1 Occupational Health, Studio Minoretti, Oggiono, ITA; 2 Biotechnology, Istituto Auxologico Italiano, Verbania, ITA; 3 Gastroenterology and Hepatology, Recep Tayyip Erdoğan University, Rize, TUR

**Keywords:** airline pilots, astronauts, aviation medicine, dysbiosis, microbiota, probiotics, space medicine

## Abstract

The human microbiota plays a crucial role in maintaining health and preventing disease; however, the effects of occupational exposure on the microbiota of aircrew and astronauts are not fully understood. This narrative review aims to synthesize the current knowledge on microbiota alterations in aerospace medicine, assess the potential of probiotics as a countermeasure, and identify key gaps that warrant further research. The references were identified through searching PubMed for English articles published between 2010 and 2024, using keywords related to microbiota, probiotics, aviation, spaceflight, pilots, and astronauts. Additionally, the bibliographies of relevant papers were reviewed. Studies in aerospace medicine were selected based on their focus on the occupational impact on microbiota and the use of probiotics in this context. For aircrew, initial studies indicate a decrease in beneficial gut bacteria, suggesting that probiotics could enhance gastrointestinal health, immunity, and overall well-being. However, unsupervised use of probiotics carries potential risks. Conversely, spaceflight induces significant changes in the gut, skin, oral, and nasal microbiota of astronauts, characterized by altered diversity and abundance of specific microbial taxa. These changes include a relative decrease in the abundance of beneficial gut bacteria, an increase in opportunistic pathogens, and evidence of microbial transfer between astronauts and spacecraft surfaces. While simulated space studies suggest the potential for probiotics to mitigate dysbiosis, direct testing done during actual spaceflight is lacking. The observed microbiota changes during spaceflight are associated with various health implications, including alterations in metabolic pathways and interactions between the microbial metabolic capabilities and the host's metabolism. In conclusion, this review highlights the profound impact of spaceflight on astronaut microbiota and the promising role of probiotics as an intervention in both space and aviation medicine. However, significant research gaps remain. These include elucidating the functional implications of microbial shifts, developing personalized countermeasures, and validating the efficacy of probiotics during spaceflight. Future studies should leverage advanced tools such as metagenomic analysis and longitudinal tracking of astronaut health to inform targeted interventions that support the well-being of aerospace personnel. Integrating data across different sites of the body and missions, facilitated by resources like the Space Omics and Medical Atlas (SOMA), can help identify consistent microbial changes induced by the unique occupational conditions of spaceflight and aviation. This integrated approach will be crucial for developing effective microbiota-based countermeasures to mitigate the occupational health risks associated with space and aviation.

## Introduction and background

The human body is host to a diverse and intricate community of microorganisms, encompassing a wide array of bacteria, fungi, archaea, and viruses. The complex interactions among these microbes and with their human host profoundly influence human health and physiology [1]. The gastrointestinal tract harbors the highest density of microorganisms, collectively referred to as the gut microbiota, which is primarily composed of Gram-positive *Firmicutes* and Gram-negative *Bacteroidetes* and plays a pivotal role in health and disease [2]. However, other anatomical sites also contain distinct microbial communities that serve important functions, such as the oral cavity, which is home to the second largest microbial community in the human body and includes *Firmicutes, Bacteroidetes, Proteobacteria, Actinobacteria,* and *Fusobacteria* [3]. The lungs, once considered sterile, contain a core microbiota consisting of *Actinobacteria, Bacteroidetes, Firmicutes*, and *Proteobacteria *[4]. The skin microbiota varies based on the microenvironment of the different regions but is generally composed of *Actinobacteria, Bacteroidetes, Cyanobacteria, Firmicutes,* and *Proteobacteria* [5]. Finally, the urinary tract harbors unique microbial communities, with a predominance of bacteria from the phylum *Firmicutes,* followed by *Actinobacteria, Bacteroidetes, and Proteobacteria* [6]. Disruption of these microbial ecosystems, known as dysbiosis [1,7], can lead to localized diseases as well as systemic effects on the host. This has garnered significant attention in several medical fields, including occupational medicine.

As individuals spend a substantial portion of their lives at work, occupational exposures may significantly impact human microbiota. In 2022, Mucci et al. [8] introduced the term "WORKbiota" to describe the effects of various occupational exposures on the human microbiota, aiming to establish a new foundation for health protection and disease prevention in workers. Within the field of occupational medicine, aeromedical science represents a highly specialized domain that integrates aviation and space medicine [9]. Aviation medicine focuses on the physiological and psychological challenges encountered by commercial airline pilots, cabin crew, military pilots, and helicopter pilots, such as circadian rhythm disruptions, sleep disturbances, and fatigue [10]. Conversely, space medicine investigates the health risks associated with spaceflight, including microgravity, cosmic radiation, confinement, and altered nutritional intake [11]. While research on microbiota alterations among airline pilots is still in its nascent stages, with limited available data [12], the field of space medicine has made significant strides in investigating the impact of spaceflight on microbiota composition [13-15]. Importantly, the gut microbiota can be modulated through the targeted administration of live beneficial bacterial strains known as probiotics [16].

This narrative review addresses the role of microbiota and the potential application of probiotics in aerospace medicine, specifically focusing on their benefits for aircrew members and astronauts. Unlike existing studies, this review uniquely examines the microbiota of airline pilots - a topic with limited research - and integrates findings across aircrew and astronauts, providing a comprehensive perspective that has not been previously addressed. Additionally, it contextualizes the use of probiotics within these occupations, an area lacking in current literature. By synthesizing diverse studies and insights, this work may offer significant implications for enhancing aviation safety and advancing human space exploration through targeted microbial management strategies.

## Review

Search strategy and selection criteria

This narrative review is based on a selective analysis of high-quality, contemporary articles on the impact of occupational exposure in the aerospace industry on the human microbiota and the potential of probiotics to mitigate these effects. The primary objective was to identify trends and enhance understanding of the current landscape of microbiota research in aerospace medicine and the potential of probiotics that confer positive benefits within an occupational health context. References for this review were identified by searching the PubMed database for peer-reviewed articles published in English between January 1, 2010, and September 1, 2024. The search terms employed included ("microbiota" AND "aviation") OR ("gut microbiota" AND "spaceflight") OR ("probiotics" AND "aviation") OR ("probiotics" AND "spaceflight") OR ("airline pilots" AND "microbiota") OR ("astronauts" AND "microbiota"). The search strategy initially identified 104 articles. These results underwent a thorough screening process to assess their relevance to the specific topics addressed in this review. Following this, the bibliographies of the selected articles were manually examined to identify any additional pertinent studies not captured in the primary search. The final reference list comprised 50 articles deemed most relevant to the topics covered in this review. These selected articles form the foundation of the evidence presented and discussed throughout the manuscript. 

Microbiota and probiotics in aviation medicine

The microbiota of airline pilots may be influenced by the highly controlled and unique aircraft cabin environment and common occupational factors associated with the profession. Several issues that have been consistently reported in aviation personnel, such as disrupted sleep patterns, fatigue, circadian rhythm disturbances, irregular meal patterns, sedentary behavior, and depressive symptoms [10], have been linked to alterations in the gut microbiota in the general population [1,2]. Weiss et al. [17] investigated aircraft microbiota by collecting 229 air and touch surface samples on ten transcontinental United States (US) flights and performed 16S rRNA sequencing. The results revealed that the airplane cabin microbiota was primarily composed of human skin and oral commensal bacteria, as well as environmentally ubiquitous bacteria [17]. A core microbiota was identified, which included operational taxonomic units within the genera *Propionibacterium, Burkholderia, Staphylococcus, and Streptococcus*. The study found distinct microbial signatures for samples from the air and touch surfaces, but there was no clear difference among individual types of touch surfaces. Additionally, the authors observed substantial beta diversity variations between flights, with no distinguishing signatures for individual flights and no consistent pattern of change in the microbial community from pre- to post-flight states [17]. A recent preliminary study conducted by our research group is the first to investigate the gut microbiota composition of airline pilots, comparing this occupational group to construction workers and fitness instructors [12]. The results revealed that airline pilots and construction workers exhibited a significantly lower abundance of health-promoting bacteria, such as *Lactobacillus *spp. and *Faecalibacterium prausnitzii*, in comparison to fitness instructors. The latter profession is known to benefit from the health-promoting effects of regular physical exercise. Furthermore, the levels of *Akkermansia muciniphila*, a bacterium closely associated with metabolic health, displayed a declining trend across the three groups, with fitness instructors showing the highest levels, followed by construction workers, and then airline pilots [12]. These findings highlight the substantial impact of the unique occupational environment and lifestyle factors on the composition of gut microbiota.

Building upon this foundation and the high number of occupational health challenges associated with commercial aviation, two studies have investigated the potential health benefits of probiotics specifically in aviation personnel [18,19]. A double-blind, randomized, placebo-controlled study examined the effects of a dietary supplement containing SYNBIO® probiotics (*Lactobacillus rhamnosus* International Microbial Center (IMC) 501® and *Lactobacillus paracasei* IMC 502®) and elderberry extract on the well-being of 40 aircrew members over a 30-day period [18]. Compared to the placebo group, the group supplemented with the probiotic-elderberry capsules demonstrated a significant increase in fecal *Lactobacillus* and *Bifidobacterium* levels, a reduction in *Enterobacteriaceae*, and higher salivary secretory immunoglobulin A concentrations [18]. Moreover, the probiotic group reported a significantly higher Psychological General Well-Being Index global score. The authors concluded that probiotic-elderberry supplementation may be beneficial for aircrew members by enhancing their gastrointestinal microbiota, immune defenses, and overall well-being when responding to the stressful conditions associated with their profession [18]. A separate open-label study focused on pilots diagnosed with *Helicobacter pylori*-negative chronic non-atrophic gastritis (CNG) and evaluated the effects of a probiotic supplement containing the yeast *Saccharomyces boulardii *and the probiotic *Enterococcus faecium* over a four-week period [19]. The results indicated that probiotic supplementation, particularly at higher doses, significantly reduced CNG symptoms such as gastric pain and bloating compared to the control group [19]. Furthermore, high-dose supplementation led to a significant increase in the pepsinogen I/II ratio, suggesting potential cytoprotective effects of the tested probiotic on the gastric mucosa. These findings highlight the potential of probiotics in alleviating upper gastrointestinal symptoms of CNG in airline pilots, offering a means to improve their gastrointestinal health [19]. However, the use of probiotics in them is not without caveats. Accordingly, we recently described a commercial airline pilot who developed brain fogginess after taking an over-the-counter probiotic supplement containing 16 strains [20]. This case should caution against the potential risks of unsupervised probiotic use, particularly in safety-sensitive professions such as commercial aviation. Table 1 provides an overview of key microbiota alterations and probiotic interventions documented in aviation medicine research.

**Table 1:** Microbiota findings and probiotic studies in airline pilots IMC: International Microbial Center; IgA: Immunoglobulin A

Aspect	Findings
Gut microbiota [12]	↓*Lactobacillus* spp., *Faecalibacterium prausnitzii, Akkermansia muciniphila*; Potential factors: Sedentary lifestyle, disrupted sleep patterns, fatigue, circadian rhythm disturbances, irregular meal patterns
Probiotic study 1 [18]	Patient group: Airline crew members; Supplement: SYNBIO® probiotics (*Lactobacillus rhamnosus* IMC 501® and *Lactobacillus paracasei *IMC 502®) with elderberry extract; Results: ↑*Lactobacillus *and *Bifidobacterium*, ↓*Enterobacteriaceae*, ↑salivary IgA, improved well-being
Probiotic study 2 [19]	Patient group: Pilots with Helicobacter pylori-negative chronic non-atrophic gastritis; Supplement: *Saccharomyces boulardii *and *Enterococcus faecium*; Results: Reduced gastric pain and bloating, ↑pepsinogen I/II ratio
Caution [20]	Risk of brain fogginess

Changes in microbial ecosystems in space medicine

While data on microbiota modifications in aviation medicine remain limited [12], there has been a growing focus on how spaceflight can alter microbial communities, particularly in light of long-term human space exploration and the future commercialization of space travel. Several recent reviews have delved into this topic [14,21-25]; however, many of the available studies have relied on animal spaceflight models or simulated space environmental factors such as microgravity, fluid shifts, and confinement, rather than focusing on the effects of spaceflight on astronauts themselves. In 2012, Saei and Barzegari [26] were the first to emphasize the pivotal role of the gastrointestinal microbiota in maintaining the health of astronauts during extended space missions and propose the development of personalized probiotic kits as a proactive strategy to bolster the resilience of the gut microbiota and mitigate health risks associated with long-duration space travel. In addressing the changes of human microbiota in astronauts, it is crucial to recognize that both animal models and simulated conditions have limitations, as they cannot fully capture the demonstrated transfer of microbes between the spacecraft and astronauts, as evidenced by Danko et al. [27] in their study of the International Space Station (ISS) environment and an astronaut's gut and oral microbiota during an extended spaceflight mission. Notably, the researchers identified certain microbial species - including *Serratia proteamaculans* and *Rickettsia australis* - that appeared to have been transferred from the ISS to the astronauts’ microbiota, with some strains persisting even after their return to Earth. Furthermore, the study suggested that the astronaut’s T-cell repertoire underwent changes to become more specific to the environmental taxa encountered on the ISS [27]. These findings underscore the complex interplay between the spacecraft environment, the astronauts’ microbiota, and the immune system. To maintain a clear emphasis on occupational medicine and avoid duplicating previous reviews that have included both simulated conditions and animal models [14,21-25], our focus will be solely on studies involving astronauts and spaceflight.

Gut Microbiota

The National Aeronautics and Space Administration (NASA) Twins Study investigated the impact of prolonged spaceflight on human physiology by comparing astronauts who were identical twins. One of them resided on the ISS for a year while the other remained on Earth as a control [28]. The study revealed several key findings related to the gastrointestinal microbiota. Throughout the study, the twin on the ground consistently exhibited greater richness of fecal microbiota compared to the twin on the ISS, although no significant differences in diversity were observed between them [28]. There was no decrease in microbiota richness or diversity in the twin on the ISS during the inflight period relative to pre- and post-flight microbiota. *Firmicutes* and *Bacteroidetes* dominated the fecal microbial communities, accounting for over 96% of sequences. The *Firmicutes* to *Bacteroidetes* ratio was elevated during the inflight period compared to pre- and post-flight levels but returned to former levels upon returning to Earth [28]. The microbial community structure in the inflight samples differed significantly from pre- and post-flight samples, a change not observed in the twin on the ground over the same period. Inflight alterations were detected in a moderate proportion (2−23%) of microbial taxa and gene content categories compared to pre/post-flight, with smaller changes observed in the twin on the ground. Spaceflight-associated dynamics were also observed in small-molecule markers of microbial metabolism, such as secondary bile acids and indole compounds [28]. In a separate study, Voorhies et al. [29] found that the gut microbiota of astronauts underwent significant alterations during six to 12-month space missions on the ISS. Shannon alpha diversity and richness increased in the gut microbiota during spaceflight and returned to pre-flight levels after returning to Earth [29]. There was a more than five-fold reduction in the relative abundance of *Akkermansia muciniphila* and *Ruminococcus* spp., and an approximately three-fold reduction in *Pseudobutyrivibrio* spp. and *Fusicatenibacter saccharivorans*. Most of these changes reverted to pre-flight levels after returning to Earth [29]. Interestingly, the gut microbiota composition became more similar across astronauts while in space, mostly due to a decrease in the abundance of a few bacterial taxa [29]. Liu et al. [15] investigated the effects of short-term spaceflight on the composition and function of the human gut microbiota in five astronauts across two missions. Metagenomic sequencing revealed that the spaceflight substantially altered the gut microbiota, with notable changes in the abundance of *Bacteroides*, *Lactobacillus*, and *Bifidobacterium *spp. [15]. The relative abundance of genes related to environmental information processing, envelope biogenesis, and glycoside hydrolases were also affected by the spaceflight. Despite these alterations, individual specificity of the gut microbiota was maintained, and most changes reverted to the pre-flight state within four weeks of returning to Earth [15]. Akinsuyi et al. [30] examined the impact of spaceflight on gut health in astronauts, focusing on gut microbiota and intestinal permeability. Metagenomic analysis demonstrated a notable reduction in beneficial, short-chain fatty acid-producing bacteria and an increase in Gram-negative pathogens, including *Citrobacter rodentium*, *Enterobacter cloacae*, *Klebsiella aerogenes*, and *Proteus hauseri*. The authors also found alterations in gene expression that pointed to increased intestinal permeability and a heightened risk of gut-related health issues during spaceflight [30].

Skin Microbiota

Voorhies et al. [29] found that the skin microbiota underwent significant alterations during ISS missions. There was a significant reduction of *Proteobacteria*, particularly *Gammaproteobacteria* and *Betaproteobacteria* spp. from the genera *Moraxella*,* Pseudomonas*, and *Acinetobacter o*n the forearm and forehead. In contrast, the relative abundance of *Firmicutes*, *Bacteroidetes* and *Actinobacteria* increased on the skin during the spaceflight along with *Streptococcus*, *Staphylococcus,* and *Corynebacterium* species. Many of these changes persisted for at least 60 days after returning to Earth [29]. The study also found a strong interaction between the skin microbiota of the astronauts and the microbial communities on the ISS surfaces [29]. Sugita et al. [31] comprehensively analyzed the temporal changes in the skin fungal microbiota of 10 astronauts before, during, and after their six-month stay at the ISS. The lipophilic fungi *Malassezia*, particularly *Malassezia restricta*, *Malassezia globosa*, and *Malassezia sympodialis*, dominated most samples, regardless of the collection period, body site, or subject [31]. During their stay on the ISS, *Malassezia* colonization increased significantly in both the cheek and chest samples. The ascomycetous yeast *Cyberlindnera jadinii *was detected in abundance in the inflight samples of five out of the 10 astronauts, suggesting the ability of this specific microorganism to proliferate in the ISS’s closed environment. Overall, fungal diversity was reduced during their stay at the ISS but recovered to pre-flight levels upon returning to Earth [31]. In a follow-up study [32], analysis of the skin mycobiota of an astronaut during a one-year stay on the ISS revealed an increased relative abundance of *Malassezia restricta* and greater levels of *Malassezia* colonization along with the presence of *Cyberlindnera jadinii* and *Candida boidinii,* which are uncommon skin mycobiota taxa.

Nasal and Oral Microbiota

In the study by Voorhies et al. [29], the nose microbiota showed similar but milder changes than the skin, with a decrease in *Gammaproteobacteria* and an increase in *Staphylococcus*, *Corynebacterium* and *Bifidobacterium *species. Notably, the tongue microbiota exhibited a reduction in *Rothia* and *Corynebacteriaceae *during the space missions [29]. Urbaniak et al. [33] examined the impact of spaceflight on the salivary microbiota of astronauts and explored potential bacterial biomarkers for viral reactivation. During the spaceflight, there was increased relative abundance of *Proteobacteria* and *Fusobacteria* while *Actinobacteria* decreased [33]. The genera *Catonella*, *Megasphaera*, and *Actinobacillus*, which were absent in more than half of the pre-flight saliva samples, were detected during the flight. Correlation analyses revealed a positive association between viral load and the relative abundance of *Gracilibacteria, Absconditabacteria*, and *Abiotrophia*, and an inverse correlation with *Oribacterium*, *Veillonella*, and *Haemophilus* [33]. Satoh et al. [34] isolated fungal strains from the nasal and pharyngeal smears and saliva of 21 astronauts pre-flight, inflight, and post-flight. When on the ground, 120 strains from 43 genera of environmental fungi were isolated from the astronauts, with *Cladosporium*, *Penicillium*, and *Aspergillus* being the dominant genera. In contrast, only 18 strains from four genera (*Aspergillus*, *Penicillium*, *Cryptococcus*, and *Rhodotorula*) were isolated from astronauts inside the ISS, indicating a significant decrease in fungal diversity during the spaceflight [34]. The number of *Candida albicans *isolates was also notably lower compared to previous data from the Apollo era [34].

Probiotics: potential for astronauts on space missions

The findings from studies on the gut, skin, nasal, and oral microbiota of astronauts during the spaceflight highlight significant alterations in microbial diversity and the relative abundance of specific taxa, raising concerns about the potential health implications for astronauts during long-duration missions [14,23,24]. In this context, the capacity of probiotics to promote a balanced gut microbiota and counteract dysbiotic changes associated with spaceflight has garnered increasing attention [35]. However, a crucial aspect that warrants careful consideration is the viability of probiotics in the unique environment of space. To address this concern, several studies have investigated the survival and efficacy of probiotics under simulated space conditions to assess their potential for use in spaceflight. Yim et al. [36] discovered that microgravity altered the physiological aspects of the probiotic *Escherichia coli* Nissle 1917 - a non-pathogenic Gram-negative strain useful for diarrhea, uncomplicated diverticular disease, and ulcerative colitis [37] - potentially compromising its efficacy relative in space. Conversely, Hwang et al. [38] identified specific lactic acid bacteria strains that enhanced immune cell proliferation and cytokine production, suggesting their potential value in maintaining immune function during the spaceflight. Furthermore, Góes-Neto et al. [39] demonstrated the ecological resilience of kombucha mutualistic community, a complex microbial ecosystem used to produce a probiotic drink, under space-like conditions. Additionally, Fajardo-Cavazos and Nicholson [40] evaluated the survival of commercial probiotics during a simulated three-year Mars mission, highlighting the robustness of spore-forming probiotics like *Bacillus subtilis*. While these studies provide valuable insights into the potential of probiotics in space (Table 2), it is important to acknowledge that they were conducted under simulated conditions and not directly on astronauts. The lack of human data from spaceflight remains a limitation that needs to be addressed through future research. Nevertheless, the current evidence suggests that carefully selected and tested probiotics may offer a promising approach to support the health of astronauts during long-duration space missions by promoting a balanced gut microbiota.

**Table 2:** Viability of probiotics under simulated space conditions

Study	Probiotic/microbial community	Findings under simulated space conditions
Yim et al. [36]	*Escherichia coli *Nissle 1917	Microgravity altered physiological aspects, potentially compromising efficacy
Hwang et al. [38]	Specific lactic acid bacteria strains	Enhanced immune cell proliferation and cytokine production
Góes-Neto et al. [39]	Kombucha mutualistic community	Demonstrated ecological resilience
Fajardo-Cavazos and Nicholson [40]	Commercial probiotics, including *Bacillus subtilis*	Spore-forming probiotics showed robustness during simulated three-year Mars mission

Discussion

Aviation Medicine

The alterations in human microbiota associated with aviation as a profession remain insufficiently elucidated. However, preliminary data indicate that airline pilots may exhibit a reduced abundance of health-promoting species in their gut microbiota, such as *Lactobacillus *spp., *Faecalibacterium prausnitzii*, and *Akkermansia muciniphila* [12]. These changes may partially stem from exposure to the specific microbial environment within airplane cabins [17], although they are more plausibly linked to occupational factors inherent to the profession. For instance, the sedentary lifestyle typical of airline pilots has been correlated with a decrease in both *Faecalibacterium prausnitzii* and *Akkermansia muciniphila* [41]. Interventions targeting gut microbiota composition, such as probiotic supplementation, hold theoretical potential for rectifying these imbalances. Although initial findings have shown promise for aircrew members [18] and pilots experiencing CNG [19], these results are preliminary and insufficient to definitively support the efficacy of probiotics in aviation medicine. Nonetheless, this area remains an intriguing avenue for further research. Probiotics have demonstrated potential benefits in addressing highly prevalent challenges among aviation professionals, including sleep disorders [42], depressive symptoms [43], and fatigue [44]. However, caution is warranted due to reports of potentially enhanced risk of brain fogginess with probiotics, as observed in a recent case report [20]. In this context, substantial efforts must be directed towards comprehensively studying the various microbiota ecosystems in pilots and aircrew members - including skin, nasal, and oral microbiota - utilizing state-of-the-art techniques. Additionally, examining both alpha diversity, which measures microbiota diversity within a single sample, and beta diversity, which assesses similarity or dissimilarity between different communities, is essential [45]. Beyond probiotics, interventions aimed at modulating microbiota in aviation personnel should also consider prebiotics and postbiotics, which are likely to present with fewer safety concerns [46]. These alternative approaches warrant thorough investigation to enhance our understanding and management of microbiota-related alterations in aviation professionals.

Space Medicine

**Figure 1:**
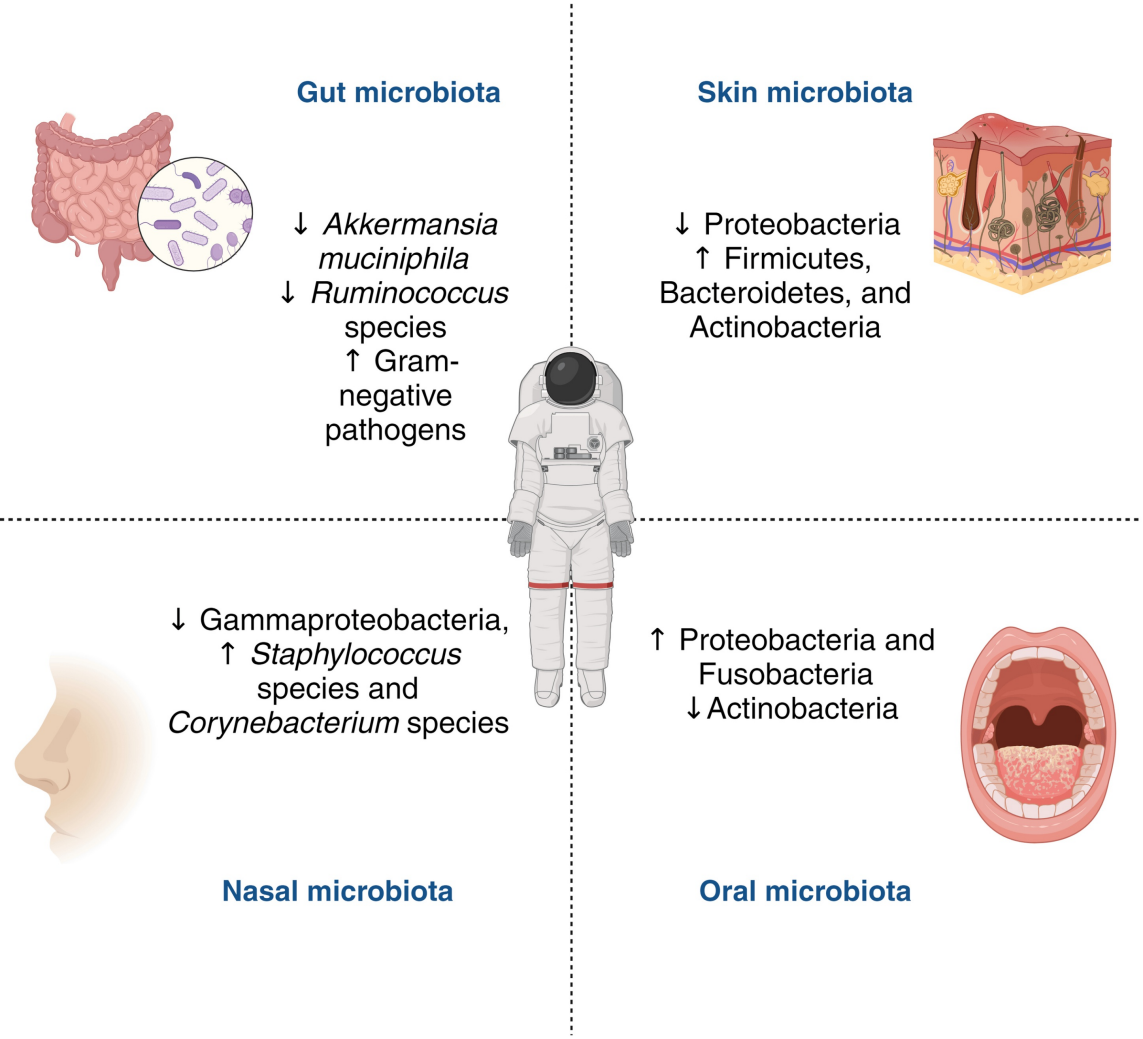
Key alterations in the human microbiota across various body sites during spaceflight missions Credit: Yusuf Yilmaz (created with biorender.com)

The study of microbiota in space medicine has progressed significantly compared to aviation medicine, offering a wealth of insights into the complex interactions between spaceflight and human microbial ecosystems. Accordingly, the available literature provides compelling evidence that spaceflight induces significant alterations in the gut [13-15], skin [22,31, 32], oral [33], and nasal [29,34] microbiota of astronauts (Figure 1).

Collectively, these findings highlight the pervasive impact of spaceflight on the microbiota across various body sites. The observed changes are characterized by shifts in microbial diversity and the relative abundance of specific taxa, which may have important implications for the astronaut's health during long-duration space missions. The gut microbiota appears to be particularly sensitive to the adverse effects of spaceflight, with substantial changes in the abundance of specific bacterial taxa, such as a decrease in beneficial short-chain fatty acid-producing bacteria and an increase in potentially pathogenic Gram-negative bacteria [14,25]. The modifications observed in the microbiota during spaceflight reveal a complex interplay between the human body, microbial communities, and the unique space environment. These alterations go beyond simple shifts in diversity and abundance, pointing to fundamental changes in microbial ecosystem dynamics [47].

**Figure 2:**
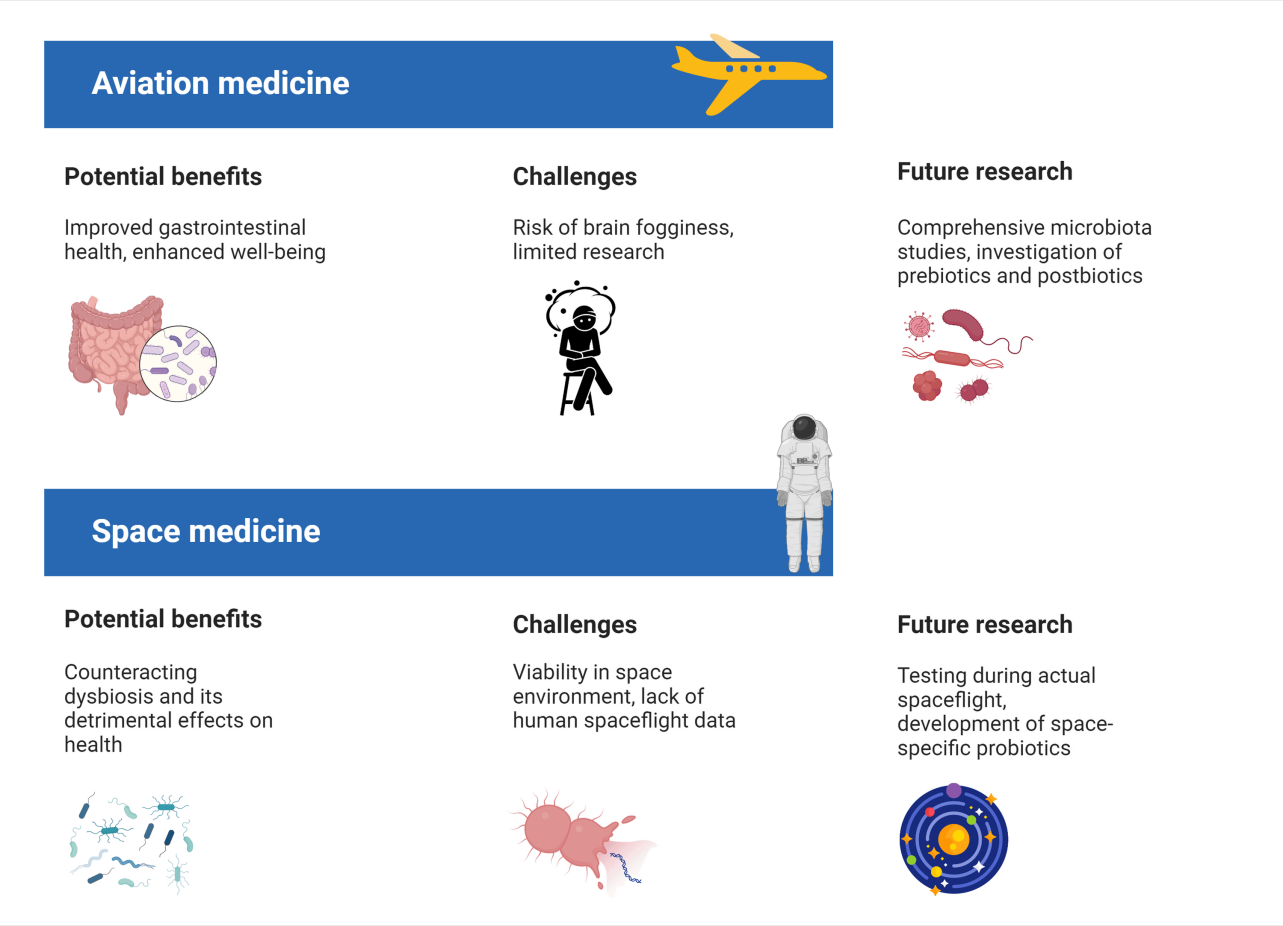
Prospective uses and key factors to consider for probiotic interventions in the field of aerospace medicine Credit: Yusuf Yilmaz (created with biorender.com)

One of the most intriguing findings is the evidence of microbial transfer between the spacecraft environment and the astronauts [27,47]. In addition, the persistence of environmental microbes suggests a level of microbial adaptation to the space environment that warrants further investigation [48]. In addition, the observed changes in the astronauts’ T-cell repertoire by becoming more specific to environmental taxa encountered on the ISS, underscore the intricate relationship between the microbiota and the immune system in space [27]. The strong interaction between skin microbiota of astronauts and surface microbial communities of the ISS suggests the potential for intentional engineering of spacecraft microbiota [27]. Creating a beneficial microbial environment within the spacecraft could potentially mitigate some of the adverse changes observed in the microbiota of astronauts. In addition, the concept of developing personalized probiotic kits for astronauts, as proposed by Saei and Barzegari [26], takes on new significance in light of the observed microbial changes. This approach could be expanded to include personalized microbial management strategies tailored to each astronaut’s unique microbiota profile and the specific challenges of their mission. In the future, the Space Omics and Medical Atlas (SOMA) can offer a valuable resource for researchers to conduct large-scale, multi-mission analyses of microbiota data from astronauts [49]. By integrating data from various body sites and spacecraft surfaces, SOMA can facilitate the identification of consistent microbial shifts induced by spaceflight and inform the development of targeted countermeasures. While the current research provides valuable insights, several challenges and areas for future investigation have emerged. The persistence of some microbial changes after returning to Earth [25,29] raises questions about the long-term health consequences of spaceflight-induced microbiota alterations. Longitudinal studies tracking the health of astronauts and their microbiota profiles over extended periods after a mission are crucial to understanding their potential impact. Future research should focus on elucidating the functional implications of the observed microbial shifts. Metagenomic and metatranscriptomic analyses could provide insights into how these changes affect metabolic pathways and host-microbe interactions, potentially informing targeted interventions [50]. Finally, the development of space-specific probiotics presents a frontier in space medicine research, offering both challenges and opportunities. While recent studies under simulated conditions have yielded promising results [35-40], the validation of probiotic efficacy during the actual spaceflight remains a critical next step. This in situ testing is essential to confirm its potential as a countermeasure against dysbiosis in the unique environment of space. Figure 2 provides a comparative overview of the current state of probiotic research in aviation and space medicine. 

Strengths and Limitations

This narrative review provides a comprehensive overview of the current state of microbiota research in aerospace medicine, covering both aviation and space medicine. By comparing the progress made in each field, the review identifies distinct challenges and opportunities, helping pinpoint gaps in knowledge and areas for future research. However, it is important to acknowledge several limitations. As a qualitative synthesis of the literature, this review may be subject to selection bias and may not provide a comprehensive assessment of all available evidence. The lack of rigorous methodologies, as employed in systematic reviews, may limit the robustness of the findings and the conclusions drawn. Another caveat is the scarcity of available data on microbiota alterations among aircrew members, restricting the depth of the analysis and the ability to draw definitive conclusions. Finally, we acknowledge that the long-term health consequences of the spaceflight-induced microbiota alterations remain unclear. 

Implications for Future Research

The findings presented in this review have several important implications for the field of aerospace medicine and the health of aircrew members and astronauts. In aviation medicine, developing specific probiotic formulations and establishing guidelines for their safe and effective use in the industry could help mitigate the adverse effects of occupational factors on the microbiota of aircrew members. For space medicine, the significant alterations observed in the gut, skin, nasal, and oral microbiota of astronauts during spaceflight underscore the importance of developing strategies to maintain microbial balance and promote overall health during long-duration missions. The evidence of microbial transfer between the spacecraft environment and astronauts suggests the potential for intentional engineering of the spacecraft microbiota to create a beneficial microbial environment. 

## Conclusions

The fields of aviation and space medicine have made progress in exploring how occupational factors may influence the human microbiota. While recent research among aircrew members remains limited, the field of space medicine has provided compelling evidence that spaceflight induces marked changes in the gut, skin, nasal, and oral microbiota of astronauts. Future research should focus on elucidating the functional implications of these microbial shifts and developing personalized microbial management strategies tailored to each astronaut’s unique microbiota profile and mission-specific challenges. Probiotics have emerged as a potential countermeasure to mitigate dysbiosis in both aviation and space medicine. Although initial findings suggest benefits, further research is needed to establish their efficacy, particularly through direct testing during the spaceflight. As the fields of aviation and space medicine continue to evolve, a deeper understanding of the complex interplay between occupational exposures, human microbiota, and health outcomes will be crucial. By unraveling these relationships and developing targeted interventions, we can work towards ensuring the well-being of those who dedicate their lives to pushing the boundaries of human exploration.
